# Size Adjustment of Zinc Oxide Nanostructures by Ultrasmall TiO_2_ Nanoparticles

**DOI:** 10.1021/acsomega.5c07829

**Published:** 2026-01-26

**Authors:** Geovanne Lemos de Assis, Artur Luis Hennemann, Helton Pereira Nogueira, Robson Raphael Guimarães, Kalil Cristhian Figueiredo Toledo, Koiti Araki

**Affiliations:** Laboratory of Supramolecular Chemistry and Nanotechnology, Department of Fundamental Chemistry, Institute of Chemistry, 28133 University of Sao Paulo. São Paulo, SP 05508-000, Brazil

## Abstract

The interaction of positively charged ZnO nanorods (*nr*-ZnO) with negatively charged ultrasmall amorphous titanium dioxide (*am*-TiO_2_) nanoparticles (NPs) induced a decrease in the *nr*-ZnO particle size while forming nanostructured core@shell *x*ZT materials (Zn/Ti molar ratios of 16, 8, 4, 2, and 1). Such an unexpected outcome was assigned to the high affinity and Zn­(II) ion adsorption capacity of *am*-TiO_2_ NP, as confirmed by the amount of Zn­(II) ion in equilibrium in solution with *am*-TiO_2_ determined by ICP-OES and the respective adsorption isotherm profile. The changes in size, morphology, and crystallinity compared to pure *nr*-ZnO were monitored by FTIR and UV–vis spectroscopy, dynamic light scattering (DLS), and X-ray diffractometry (XRD) and confirmed by transmission electron microscopy (TEM), suggesting a promising strategy to explore size-dependent nanoscale phenomena in ZnO-based nanomaterials.

## Graphical abstract

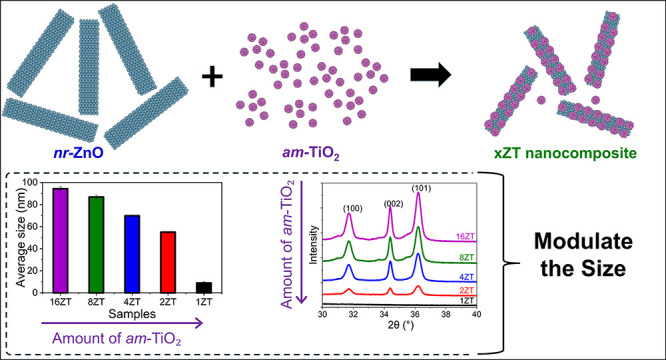

## Introduction

The ability to control both the dimensions and surface chemistry of ZnO nanocrystals is essential for tailoring their electronic, optical, and catalytic properties. Although colloidal synthesis methods offer good control over size and shape during preparation,^1–4^ achieving *postsynthetic* modulation of size and nanostructure remains a critical challenge. Strategies that enable fine adjustment of particle dimensions after synthesis are still scarce, despite their potential to unlock new functionalities in oxide-based nanomaterials.

Controlling the dimensions and the coating of ZnO nanocrystals offers a robust platform for investigating nanoscale phenomena.^1^ Colloidal synthesis methods have enabled remarkable control on size, shape, composition, and crystal structure of several inorganic nanomaterials, such as TiO_2_,^2^ gold nanoparticles,^3^ and Ni­(OH)_2_.^4^ Nevertheless, developing chemical strategies to precise postsynthesis control of both size and nanostructuration for specific applications is still challenging. Recent studies on postsynthetic size tuning of oxide nanomaterials have highlighted the importance of new strategies for fine control post synthesis. These include dissolution–reprecipitation approaches and ligand-mediated restructuring, which differ fundamentally from the Zn­(II) adsorption-driven process reported herein. In the first one, the total concentration of metal ion remains constant, but the amount available in the system after adsorption is smaller since some have been captured by an adsorbent, thus favoring the solubilization process, as described herein.

The composition, size, shape, and surface chemistry are key parameters defining the properties of nanostructured materials, and an understanding of how to control them has been eagerly pursued. For example, ZnO-based materials have been widely utilized as antimicrobial agents,^5^ in energy storage devices,^6^ dye-sensitized solar cells,^7^ light-emitting diodes (LEDs),^8^ photocatalysts,^9^ photodetectors,^10^ photodegradation of organic environmental contaminants,^11^ as well as in thermochromic materials,^12^ piezoelectric materials,^13^ gas sensors,^14^ and UV filters in cosmetic products.^15^ These properties are closely related to characteristics such as crystal structure, morphology, surface area, and crystallite size, which have been the focus of extensive research in the synthesis of ZnO nanostructures.

ZnO is an amphoteric material that exists in three polymorphic phases: rock-salt (RS), zinc blende (ZB), and hexagonal wurtzite (WZ).^16^ Under ambient conditions, only the hexagonal wurtzite phase is thermodynamically stable and exhibits the most favorable properties for the development of applications, in contrast to the RS and ZB phases. Its nanostructure and morphology can be controlled by the preparation method and respective parameters, exploiting its relatively high solubility in aqueous media,^17^ making ZnO especially suited for hydrothermal processing to obtain nanowires, nanorods, and nanobelts, exhibiting interesting piezoelectric, optical, and photocatalytic properties. They are semiconductor materials exhibiting a typical direct band gap of 3.2 eV, similar to that of anatase (3.2 eV) and larger than that of rutile (3.0 eV),^18^ but that can vary from 3.1 to 3.37 eV.^19^

Nanostructured core@shell materials can be prepared by coating nanoparticles with molecular and/or inorganic materials, generating new junctions as well as altered photocatalytic and spectroscopic properties.^20^ Generally, the interaction of carboxylate ligands and Zn­(II) ions is strong enough to anchor them onto ZnO,^21^ a material that is sparingly soluble in water but quite soluble in both acidic and strongly alkaline aqueous media given its amphoteric nature, respectively forming Zn­(II) and zincate ions.^22^ Thus, Zn­(II) ions can be more or less easily removed from the ZnO surface, thereby altering its surface chemistry, surface morphology, and roughness. Such changes are particularly relevant for photocatalytic applications, where the efficiency is strongly influenced by the active surface area and the type of exposed facets.^23^ For instance, Cheng et al.^24^ demonstrated that TiO_2_–ZnO hybrid nanostructures exhibit enhanced photocatalytic activity, attributed to changes in titanium dioxide band gap (E_g_), specific surface area, and the presence of hydroxyl groups on the nanomaterials’ surface.

Recently, we prepared readily dispersible ultrasmall amorphous titanium dioxide nanoparticles with a zeta potential as high as −40 mV,^2,25,26^ which generate very stable colloidal dispersions in aqueous media. In addition, we successfully prepared positively charged ZnO nanorods (*nr*-ZnO, ∼100 nm, zeta potential = +28 mV), which also form colloidally stable aqueous dispersions. Accordingly, herein, we report the preparation of *nr*-ZnO@*am*-TiO_2_ materials, referred to as *x*ZT, to demonstrate their size control by the stoichiometry of the precursors (*nr*-ZnO and *am*-TiO_2_) during the formation of the core@shell materials.

Hybrid ZnO–TiO_2_ nanostructures have been investigated primarily aiming the enhancement of the photocatalytic activity through heterojunction formation.^24^ In these systems, crystalline TiO_2_ phases (anatase and rutile) typically act as electron mediators. However, little attention has been given to the role of *amorphous* TiO_2_, particularly in influencing the stability and size of the ZnO particles. Recent studies have highlighted that amorphous oxides can exhibit distinct chemical reactivity and ion adsorption capability compared to their crystalline counterparts.^27–29^ These characteristics suggest that amorphous TiO_2_ may not only form core@shell architectures with ZnO but also actively modulate its size by shifting the solubility equilibrium.

Unlike previous reports on ZnO–TiO_2_ hybrid nanostructures which primarily focused on photocatalytic activity or heterojunction design, the present work demonstrates a distinctive approach in which ultrasmall amorphous TiO_2_ nanoparticles actively induce size adjustment of ZnO nanorods through Zn­(II) adsorption. This postsynthetic control of ZnO dimensions represents a novel mechanism, providing a pathway to tune ZnO properties without changing the synthesis method.

In short, herein, we demonstrate that ultrasmall amorphous TiO_2_ nanoparticles (*am*-TiO_2_, ∼4 nm) can induce the size reduction of positively charged ZnO nanorods (*nr*-ZnO) while simultaneously forming ZnO@TiO_2_ core–shell hybrids. This unexpected effect arises from the high Zn­(II) adsorption affinity of *am*-TiO_2_, which shifts the solubility equilibrium of ZnO even at neutral pH. Unlike previous approaches that rely on dissolution–reprecipitation process under acidic or alkaline conditions,^17,30,31^ our strategy enables controlled postsynthetic adjustment of ZnO nanostructures while generating heterojunctions through colloidal interactions under mild conditions, with potential implications in photocatalysis.

## Experimental Section

### Chemicals

Zinc acetate dihydrate (purity ≥98.0%), diethylene glycol (DEG), and ethanol were purchased from Labsynth, Diadema, Brazil; polyvinylpyrrolidone (PVP, MW = 40,000), and potassium bromide (KBr) were from Sigma-Aldrich, St. Louis, USA; zinc nitrate hexahydrate was from Cromoline, Diadema, Brazil; zinc sulfate heptahydrate was from Carlo Erba Reagents, Val de Reuil, France; congo red was from Givaudan, São Paulo, Brazil; P25 TiO_2_ was from Degussa, Frankfurt, Germany; and *am*-TiO_2_ (purity ≥40% TiO_2_) was kindly supplied by Spiron Tecnologia Ltd.a. All reagents were used as received without further purification. All solutions and colloidal suspensions were prepared using deionized water (DI water, resistivity ≥18.2 MΩ·cm) from a Millipore Milli-Q system.

### Synthesis of ZnO Nanorods

The colloidal suspension of ZnO nanorods (*nr*-ZnO) was prepared using a simple polyol method, as described by Lee et al.^32^ Typically, 16.0 mmol of zinc acetate, PVP (40 μmol), and 1,700 μL of deionized water were added to 40 mL of diethylene glycol, and the reaction mixture was transferred into a round-bottom flask fitted with a reflux condenser. The reaction mixture was maintained at 170 °C under vigorous stirring for 30 min and cooled to room temperature (25 °C). Then, the whitish suspension was centrifuged at 4500 rpm for 30 min and the precipitated pellet was dispersed in 40 mL of water and stored for late use. The solid sample for XRD and XPS analyses was prepared by washing the material with ethanol to remove residual DEG and drying overnight in a desiccator under a vacuum.

### Synthesis of Amorphous Titania Nanoparticles

The ∼4 nm, fully water-dispersible amorphous titania nanoparticles (*am*-TiO_2_) were prepared as a powder according to a proprietary thermal decomposition process [BR 10 2020 005909-2] and kindly provided by Spiron Tecnologia Ltd.a, São Paulo, Brazil. This product, produced in a multikilogram scale as a powder, is readily dispersible in aqueous media, generating a fully transparent dispersion, which was analyzed and subsequently used in the preparation of the *x*ZT core@shell materials.

### Synthesis of Nanostructured *x*ZT Materials

The nanostructured *x*ZT core@shell materials were prepared by rapidly adding a known amount of *am*-TiO_2_ aqueous suspension to a fixed amount of ZnO nanorod suspension at room temperature, followed by heating at 70 °C for 30 min in a water bath. This temperature was selected to accelerate ZnO–TiO_2_ interactions since at room temperature (25 °C) the same reaction requires 24 h. The relative amounts of *am*-TiO_2_ nanoparticles were varied such that the molar ratios (*x*) of *nr*-ZnO/*am*-TiO_2_ in *x*ZT are 16, 8, 4, 2, and 1.

### Affinity of *am*-TiO_2_ for Zn­(II) Ions

The adsorption profile of *am*-TiO_2_ was determined by adding 0.5, 1, 3, 5, 10, 20, 50, and 100 wt % of Zn­(II) (as nitrate, sulfate, and acetate salts) onto an aqueous suspension, waiting for 2 h to reach equilibrium at room temperature, filtering through an Amicon ultrafilter (3 kDa) by centrifugation at 4500 rpm for 5 min, and analyzing the filtrate by ICP-OES (inductively coupled plasma optical emission spectroscopy). The filtrate contains the amount of nonadsorbed zinc­(II) ions in equilibrium in solution, *C*_e_ (in ppm), allowing the determination of the amount adsorbed on *am*-TiO_2_, *q*_e_ (mg­(Zn) g^–1^(TiO_2_)), and plot of the adsorption isotherm curve. The parameters maximum adsorption capacity *Q*_max_ (mg­(Zn) g^–1^(TiO_2_)) and Langmuir constant *K*_L_ (L mg^–1^(Zn) were determined by fitting the experimental data according to the Langmuir model, using eq 1.

$$q_{e}=\frac{Q_{\max}K_{L}C_{e}}{1+K_{L}C_{e}}$$ 1

### Preparation of the Congo Red Stock Solution

A 1.0 × 10^–3^ mol L^–1^ stock solution of Congo red (MW = 696.66 g mol^–1^) was prepared by dissolving 100 μmol (69.7 mg) of the solid dye in 100 mL of deionized water (DI water) in a volumetric flask.

### Characterization Methods

#### Dynamic Light Scattering (DLS) Analysis

Samples for DLS were prepared by diluting 50 μL of the nanostructured material stock suspension in 3.0 mL of deionized water in a 10.0 mm optically path quartz cuvette. The hydrodynamic size distribution was determined using a Zetasizer Nano ZS (Malvern Instruments, United Kingdom) equipped with a red laser (λ = 632.8 nm) and a backscatter detection system positioned at 173°.

#### Zeta Potential (ZP, ζ) Analysis

Measurements were performed by transferring 1.00 mL of the sample suspension used in the DLS experiments into a polycarbonate capillary cell with gold-plated Be/Cu electrodes (DTS1070 cell) and analyzing it with a Zetasizer Nano ZS (Malvern Instruments, UK).

#### Transmission Electron Microscopy (TEM)

Samples were prepared by transferring 5.0 μL of a nanoparticle suspension (100 ppm) onto a copper grid (ultrathin C film on lacey carbon, 400 mesh; Ted Pella, Inc.), followed by drying at room temperature and subsequently in a vacuum desiccator. TEM images were acquired on a JEOL JEM-2100 microscope (Japan) operated at an accelerating voltage of 200 kV.

#### X-ray Diffraction (XRD)

Powder samples were spread on a PMMA (poly­(methyl methacrylate)) holder, and XRD patterns were collected in the 2θ range of 10–80°, with a step angle of 0.02° and an acquisition time of 0.5 s per step, using a D2 Phaser powder diffractometer (Bruker, Germany) equipped with a Cu Kα radiation source (λ = 1.54184 Å, 30 kV, 15 mA), a Lynxeye detector, and air-scatter screens to minimize diffuse scattering at low angles.

#### X-ray Photoelectron Spectroscopy (XPS)

Powder samples were mounted on carbon tape, and XPS spectra were acquired by using a Thermo Fisher Scientific K-Alpha spectrometer (UK) equipped with a hemispherical electron analyzer and a monochromatic Al Kα radiation source (1486.6 eV). Survey (full-range) and high-resolution spectra of Ti and Zn were collected with pass energies of 1000 and 100 eV, respectively. Data analysis was performed using CasaXPS software (version 2.3.25PR1.0).

#### Ultraviolet Visible (UV–Vis) Absorption Spectroscopy

Samples were prepared by diluting 20.0 μL of nanostructured materials stock suspension into 3.00 mL of DI water in a 10.0 mm optical-path quartz cuvette. Spectra were recorded in the 190–1100 nm range by using an HP 8453 diode-array spectrophotometer (Agilent, USA) equipped with deuterium and tungsten lamps as radiation sources.

#### FTIR Spectroscopy

Samples for FTIR analysis were prepared by mixing 0.5 mg of nanomaterial powder with 100.0 mg of KBr in a mortar, homogenizing with a pestle, and pressing for 5 min in a pellet die. The resulting sample discs were characterized by Fourier transform infrared spectroscopy (FTIR) using a Bruker Alpha spectrophotometer (Germany) in the 400–4000 cm^–1^ range with a resolution of 2 cm^–1^. Each spectrum represents the average of 24 scans.

#### Inductively Coupled Plasma Optical Emission Spectroscopy (ICP-OES) Analysis

The concentration of free Zn­(II) ions in equilibrium with those adsorbed on *am*-TiO_2_ was determined by ICP-OES using Agilent model 5800 equipment, analyzing the filtrate solution obtained upon filtration of Zn^2+^/*am*-TiO_2_ mixtures after 2 h equilibration time, at room temperature, through an Amicon ultrafilter, 3 kDa.

#### Photocatalytic Measurements

Samples for the analysis were prepared by adding 50 μL of Congo red stock solution (1.0 × 10^–3^ mol L^–1^) and 200 μL of a nanostructured material suspension (25 ppm) to 3.00 mL of DI water in 10 mm optical-path quartz cuvettes. The suspensions were kept in the dark for 30 min to establish adsorption–desorption equilibrium before irradiation with a sunlight simulator (Oriel Sol1A Class ABB, USA). The dye photodegradation process, as a function of time up to 150 min, was monitored using an HP 8453 diode-array spectrophotometer (Agilent, USA) equipped with deuterium and tungsten lamps over the 190–1100 nm range.

## Results and Discussion

### Physicochemical Characterization

The size distribution of the nanomaterials in water was determined by DLS, and the corresponding histograms based on particle number and scattered light intensity are shown in Figure 1A and Figure 1B, respectively, with additional data provided in Figures S1 and S2. The hydrodynamic diameters of *nr*-ZnO and *am*-TiO_2_ nanoparticles, weighted by intensity, were estimated as 85 and 4 nm, respectively. The zeta potentials (ζ) were +29.8 and −40.0 mV (Table S1, Supporting Information), indicating that the colloidal stability of both nanomaterials can be assigned to electrostatic repulsion.

**Figure 1:**
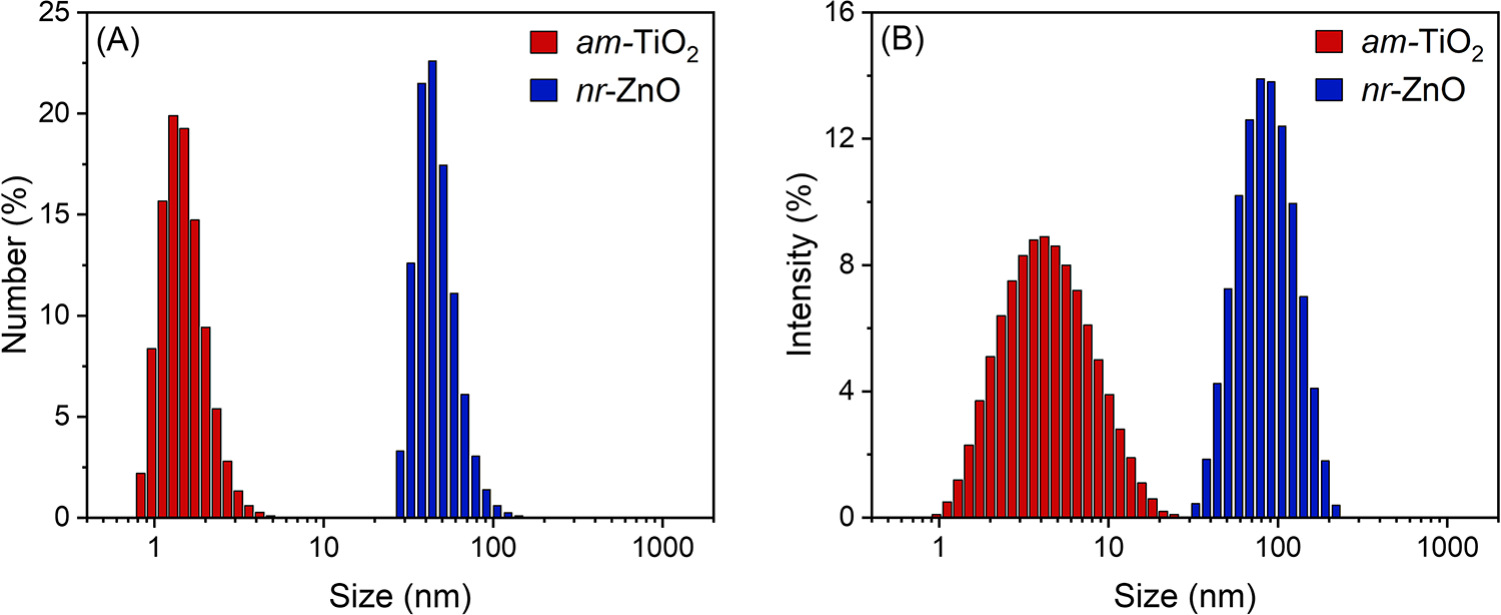
Histograms of *am*-TiO_2_ (100,000 ppm) and *nr*-ZnO (400 ppm) showing the particle size distribution weighted by (A) number and (B) intensity of scattered light.

The precise identification of the crystalline phase is another critical aspect of nanomaterials. Accordingly, X-ray diffractometry (XRD) and X-ray photoelectron spectroscopy (XPS) analyses were performed to characterize their crystalline structure and chemical composition. The *nr*-ZnO diffractogram (Figure 2A) exhibited sharp peaks at 31.7, 34.4, 36.2, 47.5, 56.6, 62.8, 66.4, 67.9, 69.0, 72.5, and 76.9° characteristic of the wurtzite phase,^33^ according to ICDD card no. 98-002-9272. Its high-resolution XPS spectrum showed the typical Zn 2p_1__/__2_ and 2p_3__/__2_ peaks at 1021.5 and 1044.6 eV, respectively, consistent with Zn­(II) in the ZnO wurtzite phase (Figure 3A).^34^ In contrast, the titanium dioxide sample exhibited no sharp peaks in the diffractogram, only two low-intensity broad features at 30 and 40° 2θ and a very broad feature at 63° 2θ, deviating from the expected diffraction profiles of anatase, rutile, or brookite—the most common crystalline phases of titanium dioxide^27–29^ (Figure 2B)—demonstrating the highly amorphous nature of *am*-TiO_2_.

**Figure 2:**
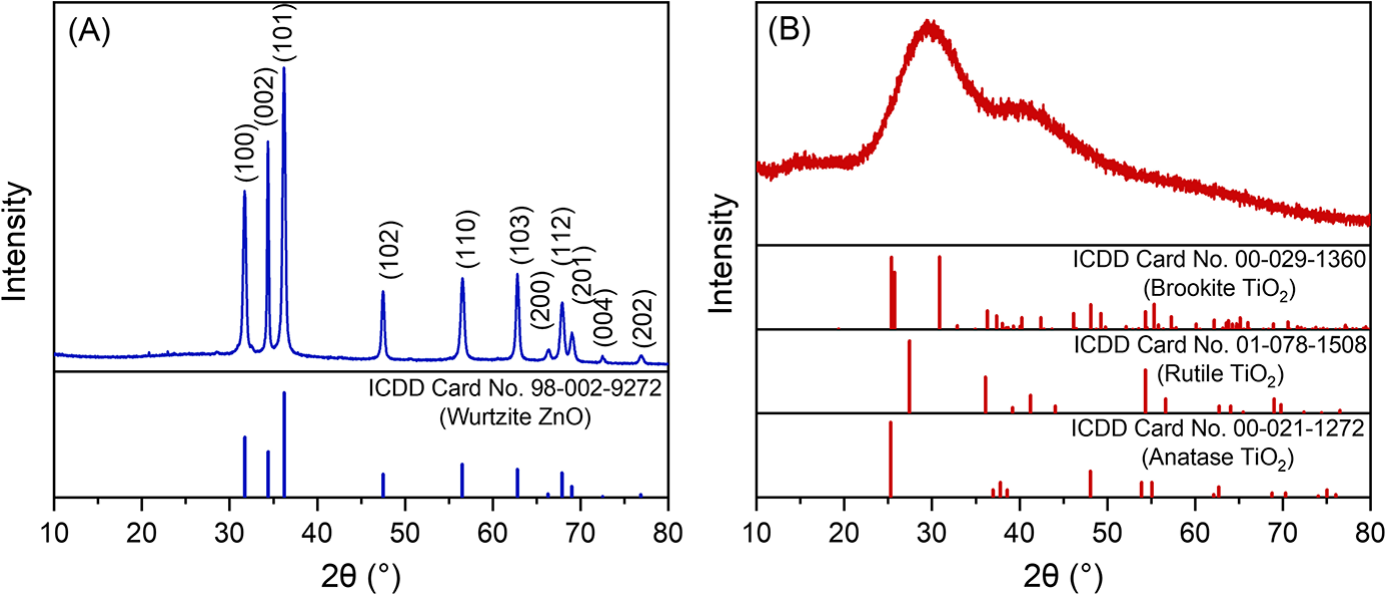
X-ray diffractograms of (A) *nr*-ZnO and (B) *am*-TiO_2_ nanoparticles compared to the respective most common crystalline phase data in the International Center for Diffraction Data, ICDD. The diffraction planes are indexed.

**Figure 3:**
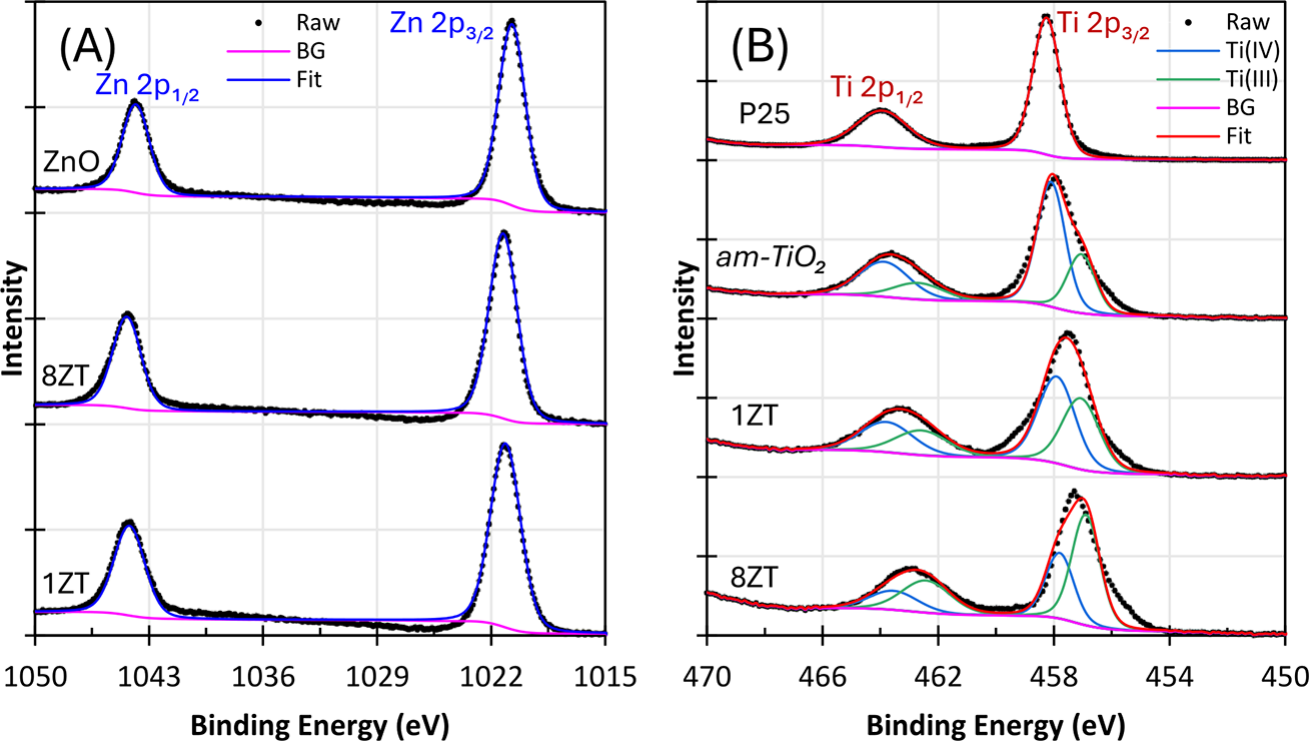
High-resolution XPS spectra of *nr*-ZnO, 8ZT, and 1ZT (A) in the Zn 2p region in comparison with ZnO and (B) in the Ti 2p region in comparison with P25 TiO_2_ and *am*-TiO_2_ nanoparticles. Experimental data (•) and the background (BG) signal (pink line).

Given their opposite electrical charges and markedly different particle sizes (42 versus 1.5 nm), we envisaged the possibility of electrostatically assembling core@shell nanomaterials with morphologies and dimensions similar to those of the ZnO nanorods. Considering the favorable electrostatic interactions, we expected the coverage of *nr*-ZnO with a layer of titanium dioxide nanoparticles, with the layer thickness increasing as a function of the relative amount of *am*-TiO_2_, until low positive to neutral zeta potentials. Accordingly, different materials were prepared by varying the amounts of *am*-TiO_2_ added to a fixed amount of ZnO to obtain *x*ZT materials (16ZT, 8ZT, 4ZT, 2ZT, and 1ZT), where *x* represents the Zn/Ti molar ratio, and the results are presented below.

The high-resolution XPS spectra of P25 TiO_2_, *am*-TiO_2_, 8ZT, and 1ZT samples are shown in Figure 3, while the survey spectra are presented in Figure S7 (SI). The commercial P25 TiO_2_ exhibits well-defined Ti 2p_1__/__2_ and Ti 2p_3__/__2_ peaks at 463.9 and 458.1 eV, respectively, perfectly matching the profile expected for Ti­(IV) species in titanium dioxide.^35^ In contrast, the corresponding XPS peaks of *am*-TiO_2_ NPs are broadened, suggesting the presence of more than one type of titanium species. In fact, the pair of peaks at 462.5 and 457.0 eV, at significantly lower binding energies than of P25 TiO_2_, could be attributed to Ti­(III) species, as expected for the high concentration of defective sites given its amorphous nature.^36^ Considering this assumption, the Ti­(III)/Ti­(IV) ratio estimated from the relative peak areas is as high as 0.47 (Table S4, Supporting Information). Interestingly, the proportion of Ti­(III) sites increases to 0.82 in 1ZT and 1.69 in 8ZT, indicating a significant rise in the Ti­(III) content when compared with pure *am*-TiO_2_. Moreover, all these nanostructured materials exhibit no absorption bands in the visible region and are white, rather than the dark or black color expected for materials with such a high concentration of Ti­(III) sites.^37^ Accordingly, the new pair of peaks revealed by deconvolution of the Ti 2p spectra is better assigned to Ti­(IV) species in different chemical environments, likely distorted Ti­(IV) sites and/or sites affected by electronic density transfer from the ZnO core. While this interpretation is consistent with the overall spectroscopic profile, complementary techniques such as EPR or Raman spectroscopy could provide additional information to confirm this assignment.

Interestingly, ZnO and *nr*-ZnO exhibit perfectly matching spectral profiles, showing the typical Zn 2p_1__/__2_ and 2p_3__/__2_ peaks at 1021.5 and 1044.6 eV,^38^ which become sharper in 8ZT and 1ZT, indicating a more homogeneous chemical environment around the Zn­(II) sites. Additional techniques were therefore employed to investigate this unexpected behavior as described below.

Good evidence of the interaction of *nr*-ZnO and *am*-TiO_2_ nanoparticles was provided by monitoring the UV–vis spectral profiles of the *x*ZT samples (Figure 4), with additional data demonstrating the reproducibility of that process shown in Figure S8. The molar ratio *x* was varied from 16 to 1, and the spectral profile was compared with those of the pure starting nanomaterials. It is important to emphasize that this spectral set corresponds to different samples prepared with the same concentration of *nr*-ZnO but with increasing concentrations of *am*-TiO_2_. Pure *nr*-ZnO exhibits an absorption onset at 385 nm and a sharp peak at 370 nm, followed by a broad absorption in the UVB region. In addition, an exponential decay of absorbance at longer wavelengths, typical of light scattering by particles in suspension, can be observed. In contrast, *am*-TiO_2_ shows only a sharp increase in absorption with onset at 345 nm (Figure 4) and no evidence of scattering in the visible range, as expected for very small fully dispersed nanoparticles and confirmed by the size distribution histogram of the nanomaterial measured by DLS.

**Figure 4:**
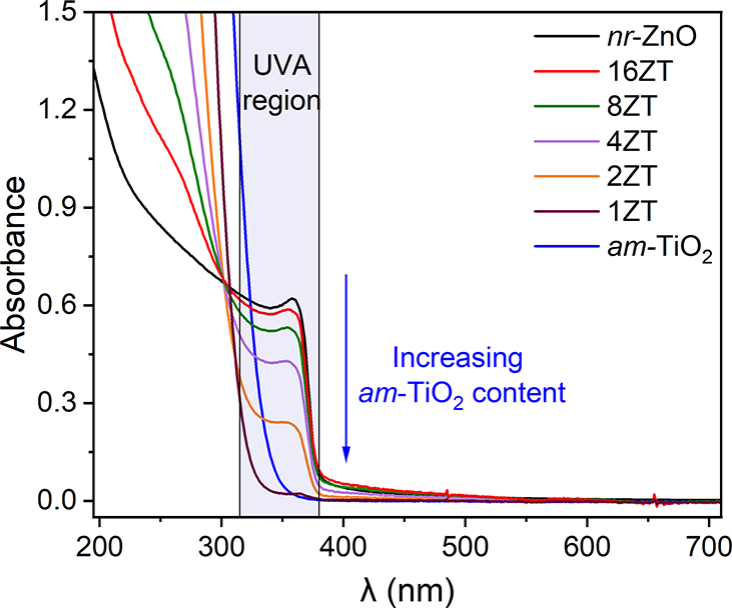
UV–vis spectrophotometric titration of a 162 ppm *nr*-ZnO aqueous suspension with increasing concentration (10–159 ppm) of *am*-TiO_2_ nanoparticles. The spectrum of pure *am*-TiO_2_ (162 ppm) in water is included for comparison.

Interestingly, as the proportion of *am*-TiO_2_ NPs increases and the Zn/Ti ratio decreases from 16 to 1, the intensity of the sharp peak at 370 nm, corresponding to the ZnO band gap absorption band,^39^ gradually decreased until it almost vanished in 1ZT. In addition, no band assignable to the Ti­(III) → Ti­(IV) intervalence transition could be observed in the visible range, demonstrating the very low concentration of Ti­(III) sites, if any. Instead, an isosbestic point, characteristic of a chemical equilibrium involving *nr*-ZnO and *am*-TiO_2_, is clearly visible at 300 nm.

To complement this qualitative discussion, Tauc plots (Figure S9, Supporting Information) were constructed for selected samples, confirming a slight band gap widening upon interaction with *am*-TiO_2_, with the estimated values increasing from 3.31 eV for pure ZnO to 3.33 eV in the *x*ZT composites. The quantitative analysis reinforces the electronic effect induced by size reduction and *am*-TiO_2_ adsorption on the nanorod surface.

What could possibly cause such dramatic changes in the intensity of the ZnO band gap transition? The UV–vis spectral profile provides a clue. Light scattering of the *x*ZT samples at 400–500 nm decreases steadily as a function of the proportion of *am*-TiO_2_ to *nr*-ZnO, that is, as *x* decreases from 16 to 1. Changes in the dimensions of the *nr*-ZnO core can influence its electronic structure, while coverage with *am*-TiO_2_ generates a junction that can alter the band gap of this semiconductor nanomaterial. A blue shift in the absorption onset compared with *am*-TiO_2_ confirms an increase in band gap energy upon formation of *x*ZT.

The band gap energies of crystalline ZnO and TiO_2_ are similar (about 3.2 eV),^40^ but the interaction increased the band gap of *am*-TiO_2_ to 3.33 eV, suggesting the formation of a blocking layer that inhibits energy and charge transfer from *nr*-ZnO in the core to species in solution. In addition, amorphous *am*-TiO_2_, with its high concentration of trapping and quenching sites, is expected to further decrease the photoactivity of TiO_2_ and, consequently, of *nr*-ZnO in the core@shell material. In short, significantly lower photocatalytic activity is expected for the *nr*-ZnO@*am*-TiO_2_ nanostructure due to inhibited charge separation and shortened lifetimes of the excited electrons and holes responsible for the photodegradation of molecules adsorbed on the *x*ZT surface. This aspect will be explored more in depth in a subsequent study.

Photocatalytic degradation assays were carried out by dispersing 1.54 ppm of *am*-TiO_2_, 8ZT, and *nr*-ZnO in water in the presence of Congo red and monitoring the kinetics of photodegradation under irradiation with a solar simulator (SI Figure S10). The amorphous TiO_2_ nanoparticles showed the highest photocatalytic activity (slope = 0.205 min^–1^), attributed to their large surface area, as shown in Figure S10 (SI), followed by *nr*-ZnO (slope = 0.063 min^–1^) and 8ZT (slope = 0.029 min^–1^). These results indicate that formation of the *nr*-ZnO/*am*-TiO_2_ interface reduces the photoactivity of both *nr*-ZnO and *am*-TiO_2_ suggesting the formation of a “barrier” for energy and/or photogenerated charge carriers, especially holes, from the core to the shell, as discussed above.

To deepen the understanding of the interaction between *nr*-ZnO and *am*-TiO_2_ in aqueous media, we investigated the structural transformation of the zinc oxide nanorods by XRD. The interaction of *nr*-ZnO with *am*-TiO_2_ NPs clearly leads to a decrease in intensity and a broadening of the ZnO diffraction peaks at 31.74, 34.40, and 36.22° as a function of the Zn/Ti ratio, as shown in Figure 5A. In fact, no diffraction peaks can be observed in the 1ZT sample, indicating the formation of an amorphous material.^27^ Considering the hypothesis that a decrease in size is responsible for this unexpected behavior, the crystallite sizes of the resulting materials were estimated based on the full width at half-maximum (fwhm) of the ZnO (100), (002), and (101) diffraction peaks. First, the fwhm values (β) were corrected according to eq 2:^41–43^

$$\beta^{2}=\beta_{s}^{2}-\beta_{r}^{2}$$ 2

in which β is the corrected fwhm value, β_s_ is the experimental fwhm, and β_r_ is the fwhm values determined for NaCl and corundum standards in the same equipment (their X-ray diffractograms are shown in Figure S5, Supporting Information). The fwhm value of the (100) peak of *nr*-ZnO was corrected using the fwhm of the (200) peak of NaCl (Δ = 0.147°) as a reference. Similarly, the fwhm values of the (002) and (101) peaks of *nr*-ZnO were corrected using the fwhm of the (104) peak of corundum (Δ = 0.105°). The crystallite size (τ) was then determined using the Scherrer equation (eq 3):^44^

$$\tau =\frac{k\lambda }{\beta \cos\left(\theta \right)}$$ 3

where *k* is Scherrer’s constant (0.9), λ is the X-ray wavelength (1.54184 Å), θ is the diffraction angle, and β is the corrected fwhm expressed in radians. The results listed in Table S3 of the Supporting Information (SI) show a decrease in ZnO crystallite size as the Zn/Ti ratio decreases, consistent with the DLS data (Figure 5B). The average size of *nr*-ZnO weighted by intensity is 85 nm, slightly smaller than the 87 nm observed for 16ZT, as expected due to its coverage by a layer of *am*-TiO_2_ NPs. Successive doubling of the *am*-TiO_2_ amount relative to *nr*-ZnO progressively decreased the *x*ZT size measured by DLS to 83, 70, 57, and ∼5 nm in 1ZT (Figure 5B). This value is close to the average *am*-TiO_2_ particle size determined by DLS suggesting that virtually only it remained, as confirmed by the absence of *nr*-ZnO characteristic diffraction peaks, but the adsorption of Zn­(II) ions on the surface (formation of Zn­(II)–*am*-TiO_2_ bonds) cannot be ruled out.

**Figure 5:**
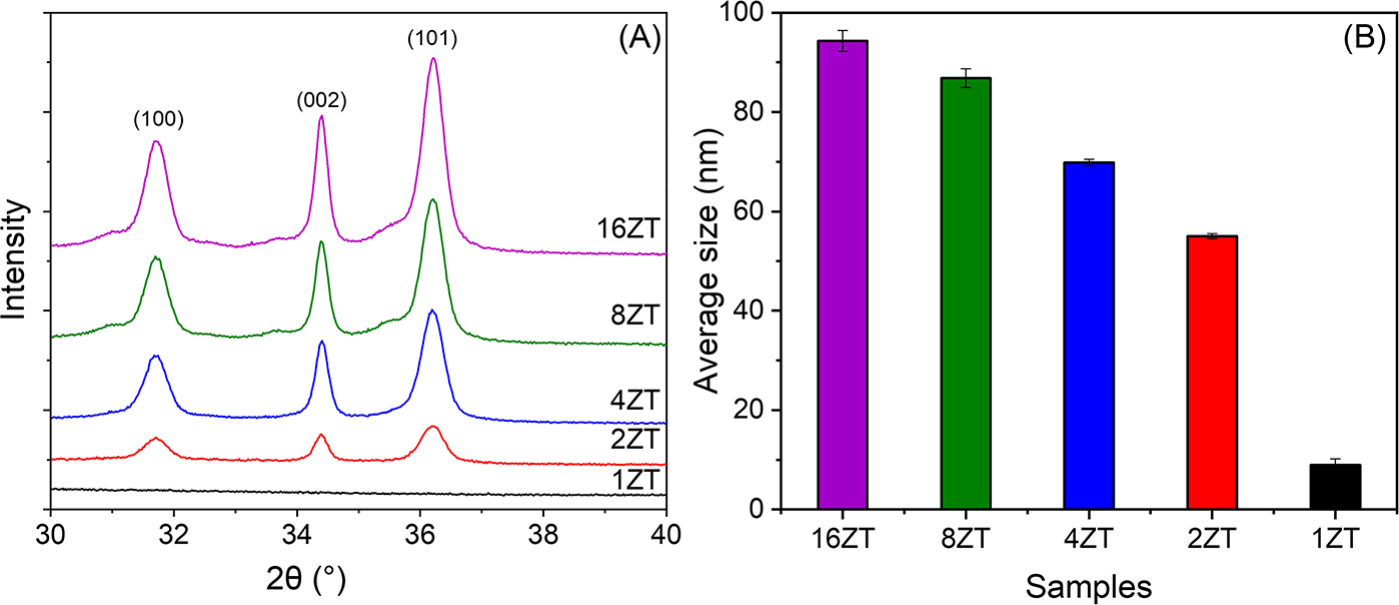
(A) X-ray diffractograms of the xZT materials (*nr*-ZnO@*am*-TiO_2_), measured in the same experimental conditions, highlighting the most intense diffraction peaks of ZnO in the nanostructures. (B) Bar graph representing *x*ZT sample average size measured by DLS weighted by the intensity of scattered light.

Finally, a more direct evaluation of the structure, morphology, and composition of *nr*-ZnO, *am*-TiO_2_, and *x*ZT materials was carried out by transmission electron microscopy (TEM). The *nr*-ZnO are predominantly constituted by 82.74 nm-long and 21.33 nm-diameter nanorods (Figure 6A,B and Figure S4A). Their 0.26 nm-spaced electron diffraction fringes observed by HRTEM (Figure 6C) are consistent with the interplanar distance between ZnO (002) planes in wurtzite nanocrystals.^45^ This result indicates that the *nr*-ZnO nanorods preferentially grew along the [001] direction (*c*-axis).^46^ In addition, the TEM images in Figure 6D and Figure S4B confirmed the DLS data, showing that *am*-TiO_2_ nanoparticles are 2.68 nm in size and amorphous. Their low degree of crystallinity was confirmed by the appearance of diffraction fringes only after focusing the electron beam on the nanoparticles, inducing local heating and crystallization, in contrast to the underfocus image shown in Figure 6D. The interaction of *am*-TiO_2_ with *nr*-ZnO in 16ZT (Figure 6E) did not significantly change the size and morphology of *nr*-ZnO but led to its coverage with a thin layer of lower-contrast material, forming random agglomerates that can be assigned to *am*-TiO_2_.

**Figure 6:**
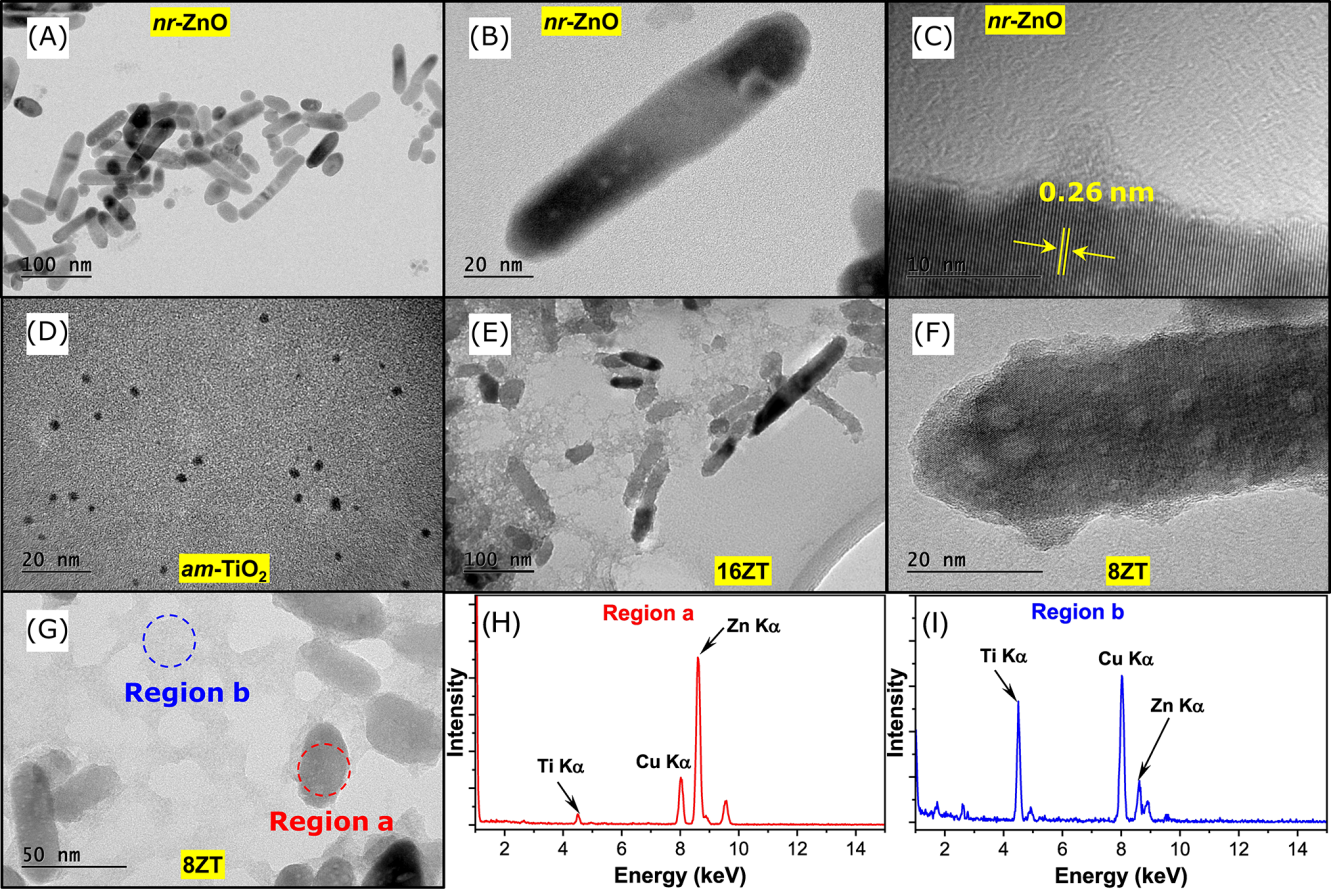
TEM images of (A–C) *nr*-ZnO, (D) *am*-TiO_2_ NPs, (E–G) *x*ZT nanocomposites, and the (H, I) EDS spectra of 8ZT.

The coverage of *nr*-ZnO (Figure 6A–C) with *am*-TiO_2_ is confirmed by the presence of a thin layer of lower-contrast material when comparing the TEM images of 8ZT (Figure 6F) with those of pure *nr*-ZnO (Figure 6B). The EDS analysis shown in Figure 6H,I was performed in regions “a” and “b” of Figure 6G, and the corresponding table of weight and atomic percentages is presented in Table S2 (SI). The first analysis carried out directly on an 8ZT nanoparticle indicated a small amount of titanium in contrast with zinc, thus confirming the formation of a core@shell structure. However, not all titanium oxide nanoparticles adhere to the *nr*-ZnO surface, despite the strong attractive electrostatic forces expected between the positively charged *nr*-ZnO (ζ = +30 mV) and the negatively charged *am*-TiO_2_ nanoparticles (ζ = −40 mV). Interestingly, the EDS analyses of the background region consistently indicated the presence of titanium as well as zinc, with a Ti/Zn ratio of 4.66, suggesting that *am*-TiO_2_ incorporates about 20% of Zn­(II) into its structure (Figure 6I, corresponding to region “b” in Figure 6G).

ZnO has low solubility in neutral pH water, such that the concentration of free Zn­(II) ions in the saturated solution tends to be quite low.^30,31^ However, *am*-TiO_2_ should be able to shift this solubility equilibrium by adsorbing Zn­(II) ions, since it induced extensive solubilization of *nr*-ZnO, as described above. TEM-EDS analyses of 1ZT, in which no ZnO nanorods could be observed at all, but only a featureless material (Figure S3A), yielded a Ti/Zn ratio of 2.56 (Figure S3C), reinforcing that hypothesis.

We note that the dramatic size reduction observed by DLS (down to ∼5 nm in 1ZT) does not fully correspond to the TEM images, which still reveal larger domains of the amorphous nanoparticles. This discrepancy occurs because DLS measures hydrodynamic sizes taking account of the molecular layer and the solvation layer, thus increasing the average size, especially at ultrasmall sizes, while TEM is mainly sensitive to the electronically dense crystalline oxide core of nanoparticles.

Accordingly, the adsorption isotherm profile of Zn­(II) on *am*-TiO_2_ nanoparticles (Figure 7) was determined by measuring the amount of Zn­(II) ion in equilibrium with the nanoparticle suspension present in the solution obtained after filtration through a 3 kDa Amicon ultrafilter. The linear increase in the amount of adsorbed Zn­(II) ions compared to the free ion in solution indicates a significantly high affinity of *am*-TiO_2_ at low *C*_e_ values. Overall, the adsorption isotherm presented a typical saturation profile and could be fitted by the Langmuir model (eq 1) using a *Q*_max_ = 734 mg g^–1^ (TiO_2_ and *K*_L_ = 0.008 L mg^–1^). This behavior can be attributed to its adsorption by electrostatic interaction with highly negatively charged *am*-TiO_2_ nanoparticles. Nevertheless, binding of Zn­(II) ions to specific coordination sites on the nanoparticle surface cannot be ruled out, particularly given their amorphous nature and high adsorption capacity. No significant effect of the acetate, nitrate, or sulfate counter-anions was observed.

**Figure 7:**
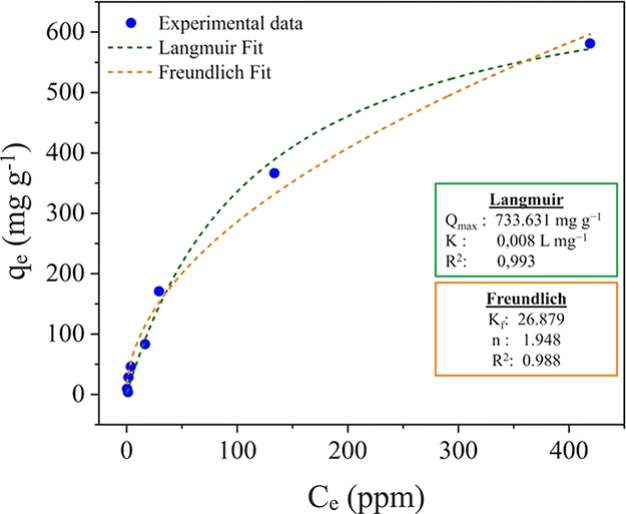
Graphs of the concentration of adsorbed Zn­(II) ion (blue circles) as a function of the free ion in equilibrium in solution determined by ICP-OES analysis of the filtrate solution obtained by filtration through a 3 kDa Amicon ultrafilter. The fitting curves according to the Langmuir and Freundlich model (green and orange dashed line) are included.

Interestingly, crystalline TiO_2_, such as anatase and rutile, is not known to adsorb such large amounts of Zn­(II) ions or to have the capacity to remove them from the surface of crystalline ZnO particles. Nevertheless, the adsorption capacity is known to be enhanced by decreasing particle size and increasing surface area, while the affinity can be further increased by the formation of defective sites on the surface. It should be noted that *am*-TiO_2_ nanoparticles are ultrasmall (∼3 nm) and amorphous, which enhances their adsorption capacity and reinforces the unique role of the amorphous state in increasing the Zn­(II) affinity. In addition, possible contributions from electrostatic effects given their high negative zeta potential, together with dangling carboxylate groups from citrate ligands (Figure S6 in the Supporting Information), cannot be ruled out.

In fact, the wavenumbers and intensities of the carboxylate bands at 1620 and 1399 cm^–1^, respectively, assigned to the asymmetric and symmetric stretching modes of the carboxylate groups^47^ of the citrate ligands on *am*-TiO_2_, are sensitive to the chemical environment and coordination of metal ions. The shift of the carboxylate asymmetric stretching mode from 1620 to 1577 cm^–1^ observed in the FTIR spectra of *x*ZT materials can thus be attributed to coordination with Zn­(II) ions, as reflected by the increase in the 1577 cm^–1^/1620 cm^–1^ ν_as_-COO vibrational mode peak intensity ratio from 0.146 in *am*-TiO_2_ to 0.316 in 2ZT, thus confirming the contribution of citrate ligands. The broad band at 3400 cm^–1^ was attributed to the stretching vibrational mode of hydroxyl groups (ν­(O–H)) present on the nanomaterials, as well as to small amounts of adsorbed water molecules.^48^ Therefore, the citrate ligands play a dual role, providing colloidal stability to *am*-TiO_2_ while enhancing its capacity to sequester Zn­(II) ions from ZnO.

The shift of the carboxylate asymmetric stretching band from 1620 cm^–1^ in *am*-TiO_2_ to 1577 cm^–1^ in 2ZT indicates the coordination of Zn­(II) ions by citrate ligands on the TiO_2_ surface. This observation, together with the increase in the 1577/1620 cm^–1^ intensity ratio, provides additional evidence for Zn­(II) removal from ZnO and subsequent adsorption onto *am*-TiO_2_, whereas crystalline TiO_2_ phases, such as anatase and rutile, do not adsorb Zn­(II) ions strongly enough to remove them from the ZnO surface, confirming the unique role of the amorphous state. These findings support the proposed dissolution–adsorption mechanism and confirm the evidence gathered by DLS, EDS, and XPS analyses. The size reduction induced by *am*-TiO_2_ proposed herein is conceptually different from that of dissolution–precipitation since it is induced by an actual reduction of the Zn­(II) ions in solution in equilibrium with *nr*-ZnO, thus shifting its solubility equilibrium to the right given the amount of Zn­(II) that has been captured by *am*-TiO_2_, while the total amount of Zn­(II) remains more or less constant in the dissolution/reprecipitation process.

## Conclusions

Positively charged ZnO nanorods (*nr*-ZnO, ZP = +30 mV) and negatively charged titanium dioxide nanoparticles (*am-TiO*_*2*_, ZP = −40 mV) were prepared and characterized, revealing their ultrasmall amorphous nature and high Zn­(II) affinity. Their electrostatically driven interaction led to the formation of core@shell *x*ZT nanostructures and, unexpectedly, also decreased the *nr*-ZnO particle size. The solubilization of ZnO induced by *am*-TiO_2_, by shift of solubility equilibrium through Zn­(II) ion removal from solution, was confirmed by ICP-OES. Samples were prepared by interacting *am*-TiO_2_ with Zn­(II) and analyzing its concentration in the solution obtained upon filtration through a 3 kDa Amicon ultrafilter. The decrease in size was monitored by DLS, TEM, and XRD, with peak intensities steadily decreasing as the Zn/Ti molar ratio decreased (*x* = 16, 8, 4, 2, 1). These structural changes correlated with the intensity of the band gap transition at 370 nm, reflecting alterations in the electronic properties of ZnO nanorods. In short, the ultrasmall size and amorphous nature of *am*-TiO_2_ confer high affinity and adsorption capacity for Zn­(II) ions, shifting the solubility equilibrium of ZnO thus enabling postsynthetic adjustment of the *x*ZT core@shell nanomaterial size as a function of the *am*-TiO_2_/*nr*-ZnO ratio while generating heterojunctions. These findings suggest a promising strategy for exploring size- and surface-chemistry–dependent nanoscale phenomena in ZnO-based nanomaterials that can improve charge separation and photocatalytic activity.

## Supplementary Material



## Data Availability

The data supporting this article have been included as part of the Supporting Information.

## Notes

Dynamic light scattering (DLS) graphics, UV–vis spectra, transmission electron microscopy (TEM) images and size distribution analysis, energy-dispersive X-ray apectroscopy (EDS), X-ray diffractometry (XRD), FTIR spectra, Tauc plot method, and tables with zeta potential, EDS, XRD, X-ray photoelectron spectroscopy (XPS) parameters, and photodegradation curves (PDF)

## References

[1] Mehra S., Chan E. M., Salleo A. (2015). Modular synthetic design enables
precise control of shape and doping in colloidal zinc oxide nanorods. J. Mater. Chem. C.

[2] Hennemann A. L., Nogueira H. P., Ramos M. D., Correra T. C., Hennemann B. L., Araki K. (2024). Amorphous Titanium Dioxide Nanoparticles
and Their Unexpected Fragmentation in MALDI-TOF/MS. ACS Omega.

[3] Daniel M. C., Astruc D. (2004). Gold nanoparticles:
assembly, supramolecular chemistry,
quantum-size-related properties, and applications toward biology,
catalysis, and nanotechnology. Chem. Rev..

[4] Rocha M. A., Winnischofer H., Araki K., Anaissi F. J., Toma H. E. (2011). A new insight
on the preparation of stabilized alpha-nickel hydroxide nanoparticles. J. Nanosci. Nanotechnol.

[5] Babayevska N., Przysiecka Ł., Iatsunskyi I., Nowaczyk G., Jarek M., Janiszewska E., Jurga S. (2022). ZnO size and shape effect on antibacterial
activity and cytotoxicity profile. Sci. Rep.

[6] Bishwakarma H., Tyagi R., Kumar N., Das A. K. (2023). Green synthesis
of flower shape ZnO-GO nanocomposite through optimized discharge parameter
and its efficiency in energy storage device. Environ. Res..

[7] Anta J. A., Guillén E., Tena-Zaera R. (2012). ZnO-Based
Dye-Sensitized Solar Cells. J. Phys. Chem. C.

[8] Pearton S. J., Ren F. (2014). Advances in ZnO-based
materials for light emitting diodes. Curr. Opin.
Chem. Eng..

[9] Mgolombane M., Majodina S., Bankole O. M., Ferg E. E., Ogunlaja A. S. (2021). Influence
of surface modification of zinc oxide–based nanomaterials on
the photocatalytic reduction of carbon dioxide. Mater. Today Chem..

[10] Deka
Boruah B. (2019). Zinc oxide ultraviolet photodetectors: rapid progress from conventional
to self-powered photodetectors. Nanoscale Adv..

[11] Manikanika, Chopra L. (2022). Photocatalytic activity
of zinc oxide for dye and drug degradation: A review. Mater. Today Proc..

[12] Ito H., Yoshioka D., Hamada M., Okamoto T., Kobori Y., Kobayashi Y. (2022). Photochromism of colloidal ZnO nanocrystal powders
under ambient conditions. Photochem. Photobiol.
Sci..

[13] Srikanth, K. S. ; Wazeer, A. ; Mathiyalagan, P. ; Vidya, S. ; Rajput, K. ; Kushwaha, H. S. Piezoelectric properties of ZnO. In Nanostructured Zinc Oxide, Awasthi, K. , Ed. Elsevier: 2021; pp 717–736.

[14] Kang Y., Yu F., Zhang L., Wang W., Chen L., Li Y. (2021). Review of
ZnO-based nanomaterials in gas sensors. Solid
State Ionics.

[15] Fajzulin I., Zhu X., Möller M. (2015). Nanoparticulate inorganic UV absorbers:
a review. J. Coat. Technol. Res..

[16] Raha S., Ahmaruzzaman M. (2022). ZnO nanostructured
materials and their potential applications:
progress, challenges and perspectives. Nanoscale
Adv..

[17] Elhaj
Baddar Z., Matocha C. J., Unrine J. M. (2019). Surface coating
effects on the sorption and dissolution of ZnO nanoparticles in soil. Environ. Sci.: Nano.

[18] Janczarek M., Kowalska E. (2017). On the Origin of Enhanced
Photocatalytic Activity of
Copper-Modified Titania in the Oxidative Reaction Systems. Catalysts.

[19] Davis K., Yarbrough R., Froeschle M., White J., Rathnayake H. (2019). Band gap engineered
zinc oxide nanostructures via a sol–gel synthesis of solvent
driven shape controlled crystal growth. RSC
Adv..

[20] Ghosh
Chaudhuri R., Paria S. (2012). Core/Shell Nanoparticles: Classes,
Properties, Synthesis Mechanisms, Characterization, and Applications. Chem. Rev..

[21] Das S., Dutta K., Pramanik A. (2013). Morphology
control of ZnO with citrate:
a time and concentration dependent mechanistic insight. CrystEngComm.

[22] Krężel A., Maret W. (2016). The biological
inorganic chemistry of zinc ions. Arch. Biochem.
Biophys..

[23] Gouveia A. F., Lemos S. C. S., Leite E. R., Longo E., Andrés J. (2023). Back to the
Basics: Probing the Role of Surfaces in the Experimentally Observed
Morphological Evolution of ZnO. Nanomaterials.

[24] Cheng C., Amini A., Zhu C., Xu Z., Song H., Wang N. (2014). Enhanced photocatalytic performance of TiO2-ZnO hybrid nanostructures. Sci. Rep..

[25] Kawassaki R. K., Romano M., Klimuk Uchiyama M., Cardoso R. M., Baptista M. S., Farsky S. H. P., Chaim K. T., Guimarães R. R., Araki K. (2023). Novel Gadolinium-Free Ultrasmall Nanostructured Positive Contrast
for Magnetic Resonance Angiography and Imaging. Nano Lett..

[26] França
Dias M., Ken Kawassaki R., Amaral de Melo L., Araki K., Raphael Guimarães R., Ligorio
Fialho S. (2025). Optimizing Retinal Imaging: Evaluation of ultrasmall TiO2 nanoparticle-
fluorescein conjugates for improved Fundus Fluorescein Angiography. Methods.

[27] Sun S., Song P., Cui J., Liang S. (2019). Amorphous TiO2 nanostructures:
synthesis, fundamental properties and photocatalytic applications. Catal. Sci. Technol..

[28] Lehutso R. F., Tancu Y., Maity A., Thwala M. (2021). Characterisation of
Engineered Nanomaterials in Nano-Enabled Products Exhibiting Priority
Environmental Exposure. Molecules.

[29] Nimmy A. V., Mahesh A., Anandakumar V. M., Biju V. (2024). Revealing the role
of defect-induced trap levels in sol–gel-derived TiO2 samples
and the synergistic effect of a mixed phase in photocatalytic degradation
of organic pollutants. J. Phys. Chem. Solids.

[30] Leung C. Y., Tu Y., Tang B. Z., Wang W.-X. (2019). Dissolution kinetics of zinc oxide
nanoparticles: real-time monitoring using a Zn2+-specific fluorescent
probe. Environ. Sci.: Nano.

[31] David C. A., Galceran J., Rey-Castro C., Puy J., Companys E., Salvador J., Monné J., Wallace R., Vakourov A. (2012). Dissolution
Kinetics and Solubility of ZnO Nanoparticles Followed by AGNES. J. Phys. Chem. C.

[32] Lee S., Jeong S., Kim D., Hwang S., Jeon M., Moon J. (2008). ZnO nanoparticles with
controlled shapes and sizes prepared using
a simple polyol synthesis. Superlattices Microstruct..

[33] Sugihartono I., Dianisya D., Isnaeni I. (2018). Crystal structure analyses of ZnO
nanoparticles growth by simple wet chemical method. IOP Conf. Ser.: Mater. Sci. Eng..

[34] Al-Gaashani R., Radiman S., Daud A. R., Tabet N., Al-Douri Y. (2013). XPS and optical
studies of different morphologies of ZnO nanostructures prepared by
microwave methods. Ceram. Int..

[35] Wang T., Li Y., Pan J. h., Zhang Y. l., Wu L. g., Dong C. y., Li C. j. (2019). Alcohol solvothermal reduction for commercial P25 to harvest weak
visible light and fabrication of the resulting floating photocatalytic
spheres. Sci. Rep..

[36] Bharti B., Kumar S., Lee H. N., Kumar R. (2016). Formation of oxygen
vacancies and Ti3+ state in TiO2 thin film and enhanced optical properties
by air plasma treatment. Sci. Rep..

[37] Li G., Lian Z., Li X., Xu Y., Wang W., Zhang D., Tian F., Li H. (2015). Ionothermal
synthesis
of black Ti3+-doped single-crystal TiO2 as an active photocatalyst
for pollutant degradation and H2 generation. J. Mater. Chem. A.

[38] Heng C. L., Zhao C. N., Zhang L., Xiang W., Su W. Y., Yin H. X., Gao Y. K., Yin P. G., Finstad T. G. (2020). Effects
of Yb doping on the structure and near band-edge emission of ZnO thin
films on Si after high temperature annealing. J. Lumin..

[39] Tarish S., Al-Haddad A., Xu R., Cao D., Wang Z., Qu S., Nabi G., Lei Y. (2016). The shift of the optical absorption
band edge of ZnO/ZnS core/shell nanotube arrays beyond quantum effects. J. Mater. Chem. C.

[40] Navidpour A. H., Abbasi S., Li D., Mojiri A., Zhou J. L. (2023). Investigation
of Advanced Oxidation Process in the Presence of TiO2 Semiconductor
as Photocatalyst: Property, Principle, Kinetic Analysis, and Photocatalytic
Activity. Catalysts.

[41] Rodrigues L. C. V., Stefani R., Brito H. F., Felinto M. C. F. C., Hölsä J., Lastusaari M., Laamanen T., Malkamäki M. (2010). Thermoluminescence
and synchrotron radiation studies on the persistent luminescence of
BaAl2O4:Eu2+,Dy3+. J. Solid State Chem..

[42] Antony
Lilly Grace M., Veerabhadra Rao K., Anuradha K., Judith Jayarani A., Arun kumar A., Rathika A. (2023). X-ray analysis and size-strain plot
of zinc oxide nanoparticles by Williamson-Hall. Mater. Today Proc..

[43] Weidenthaler C. (2011). Pitfalls in
the characterization of nanoporous and nanosized materials. Nanoscale.

[44] Patterson A. L. (1939). The Scherrer
Formula for X-Ray Particle Size Determination. Phys. Rev..

[45] Chang J. S., Strunk J., Chong M. N., Poh P. E., Ocon J. D. (2020). Multi-dimensional
zinc oxide (ZnO) nanoarchitectures as efficient photocatalysts: What
is the fundamental factor that determines photoactivity in ZnO?. J. Hazard. Mater..

[46] Idiawati R., Mufti N., Taufiq A., Wisodo H., Laila I. K. R., Fuad A., Sunaryono (2017). Effect of Growth Time on the Characteristics
of ZnO
Nanorods. IOP Conf. Ser.: Mater. Sci. Eng..

[47] Papageorgiou S. K., Kouvelos E. P., Favvas E. P., Sapalidis A. A., Romanos G. E., Katsaros F. K. (2010). Metal–carboxylate interactions
in metal–alginate complexes studied with FTIR spectroscopy. Carbohydr. Res..

[48] Perakis F., De Marco L., Shalit A., Tang F., Kann Z. R., Kühne T. D., Torre R., Bonn M., Nagata Y. (2016). Vibrational
Spectroscopy and Dynamics of Water. Chem. Rev..

