# Supplementary material for: Size Adjustment of Zinc Oxide Nanostructures by Ultrasmall TiO2 Nanoparticles
Source: ACS Omega. 2026 Jan 26;11(5):7303–12. doi: 10.1021/acsomega.5c07829 (PMC12903040; doi:10.1021/acsomega.5c07829)
Supplement: Supplementary file 1 [file ao5c07829_si_001.pdf]

Supplementary Information

Size Adjustment of Zinc Oxide Nanostructures by Ultrasmall TiO_2 Nanoparticles

Geovanne Lemos de Assis,*[a] Artur Luis Hennemann,[a] Helton Pereira Nogueira,[a] Robson Raphael Guimarães,[a] Kalil Cristhian Figueiredo Toledo[a] and Koiti Araki*[a]

[a.] *Laboratory of Supramolecular Chemistry and Nanotechnology, Department of Fundamental Chemistry, Institute of Chemistry, University of São Paulo. 05508-000 São Paulo, SP, Brazil.*

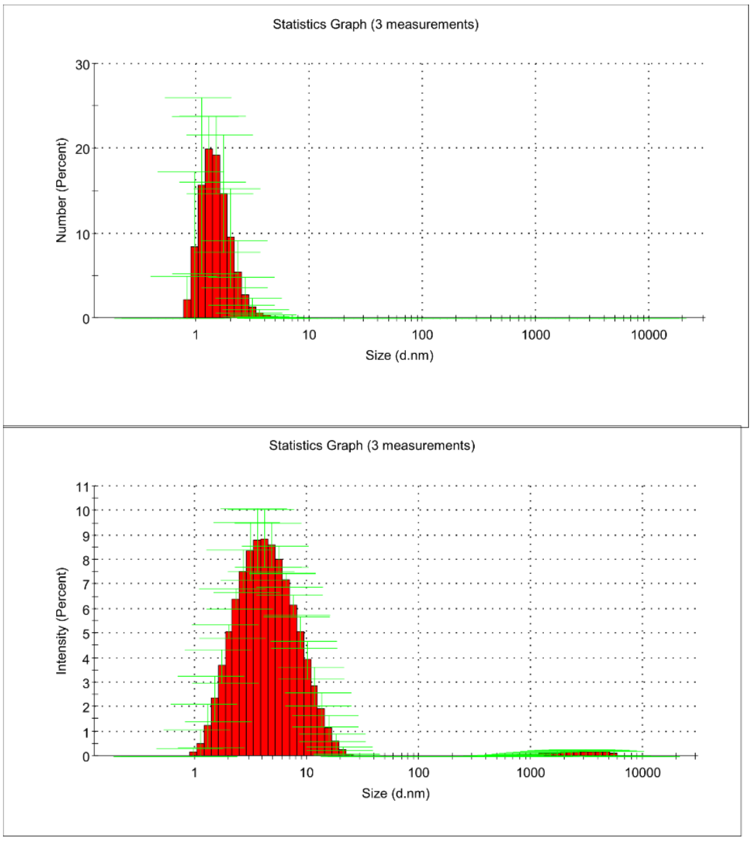

Figure S1. Histograms of the particle size distribution of *am*-TiO_2, weighted by number and by intensity of scattered light.

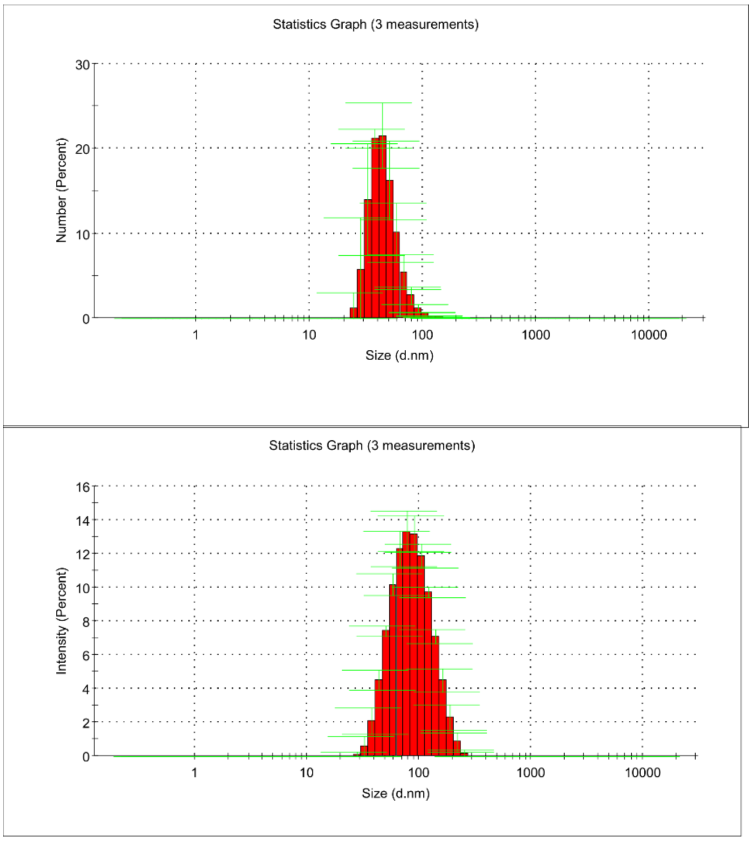

Figure S2. Histograms of the particle size distribution of *nr*-ZnO, weighted by number and by intensity of scattered light.

Table S1. Average zeta potentials and standard deviations of the nanocomposites.

Samples	Zeta Potential (mV)	Standard Deviation
***nr*-ZnO**	29.77	0.12
**16Z**	-23.1	0.71
**8ZT**	-37.1	0.41
**4ZT**	-41.6	0.66
**2ZT**	-41.6	0.79
**1ZT**	-40.7	0.92
***am*-TiO_2**	-40.0	1.95

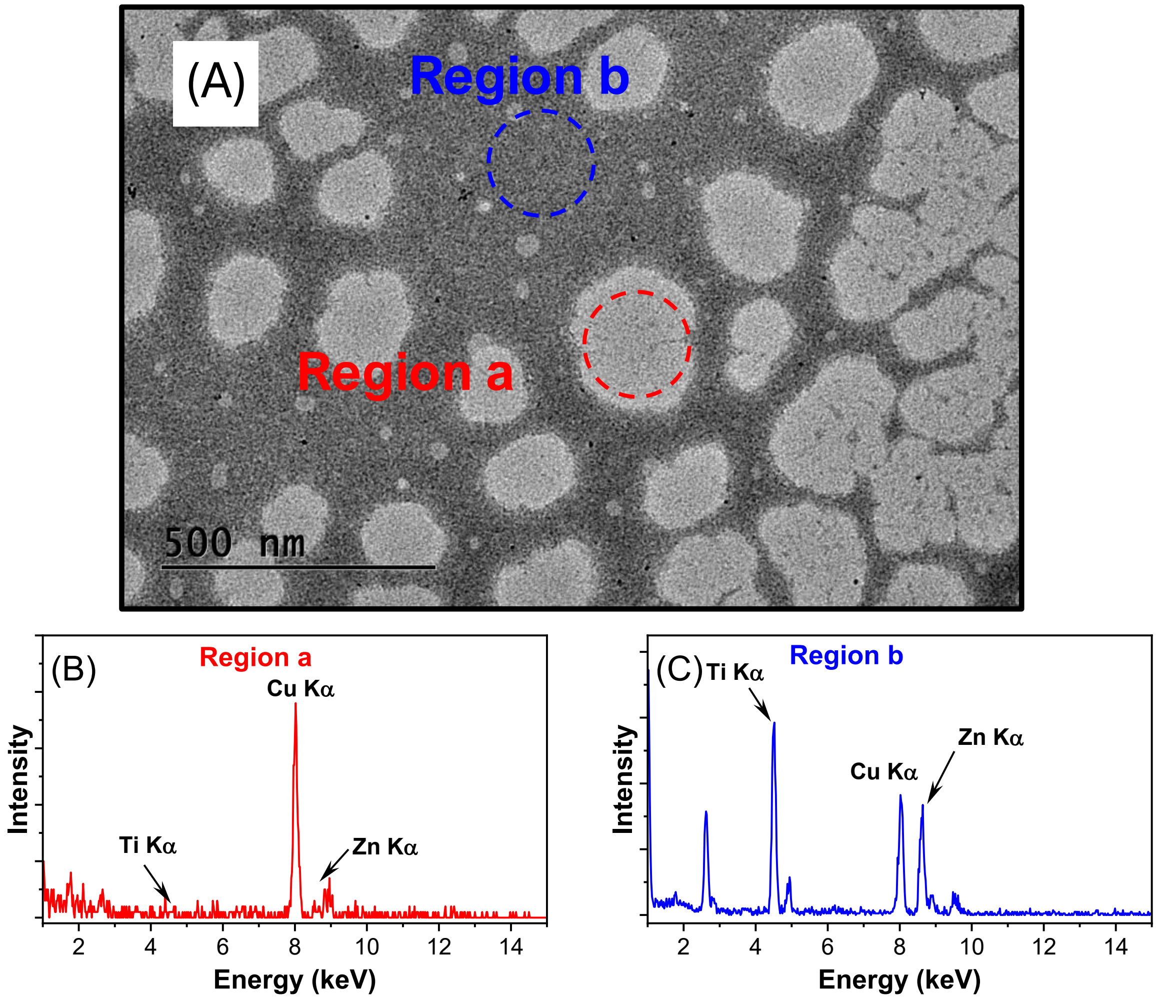

Figure S3. TEM image of 1ZT (A) highlighting regions 'a' and 'b'; their corresponding EDS spectra are presented in (B) and (C), respectively.

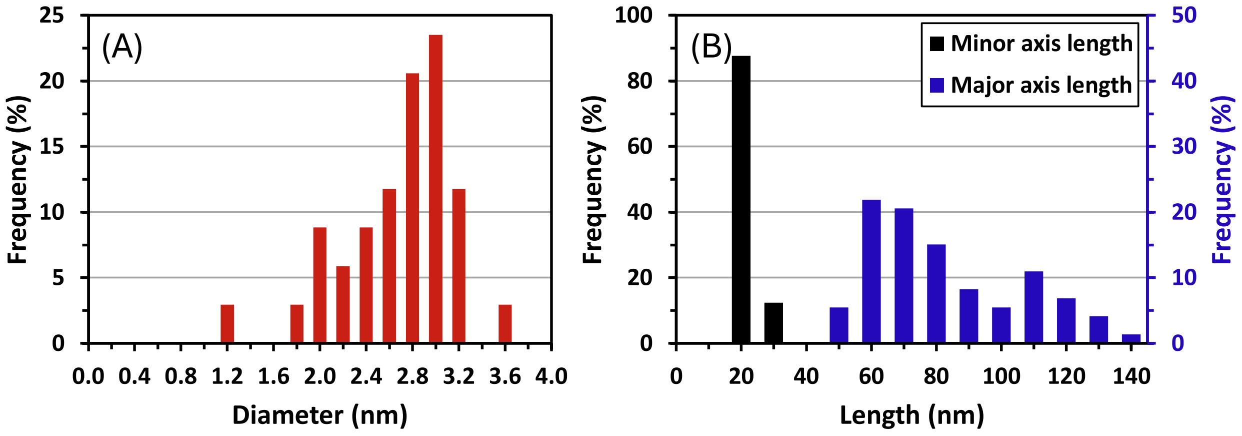

Figure S4. Size distribution analysis of (A) *am*-TiO_2 nanoparticles and (B) ZnO nanorods.

Table S2. Weight and atomic percentages of Ti, and Zn obtained from EDS analysis of representative xZT composite samples.

Samples		Element	Weight (%)	Atomic (%)
**8ZT**	Region a	**Ti**	4.85	6.51
		**Zn**	95.15	93.49
	Region b	**Ti**	77.32	82.32
		**Zn**	22.68	17.68
**1ZT**	Region a	**Ti**	Not detected	
		**Zn**	Not detected	
	Region b	**Ti**	65.22	71.92
		**Zn**	34.78	28.08

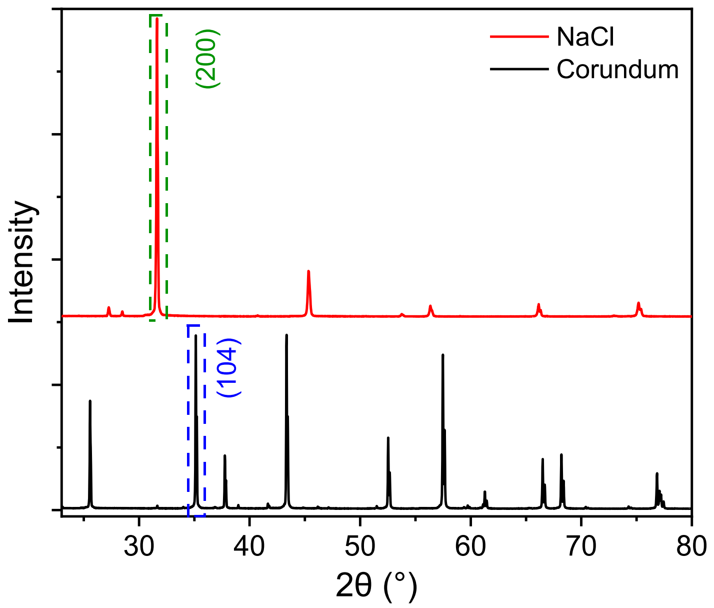

Figure S5. X-ray Diffraction Patterns of NaCl and Corundum (Al_2O_3) used as reference standards.

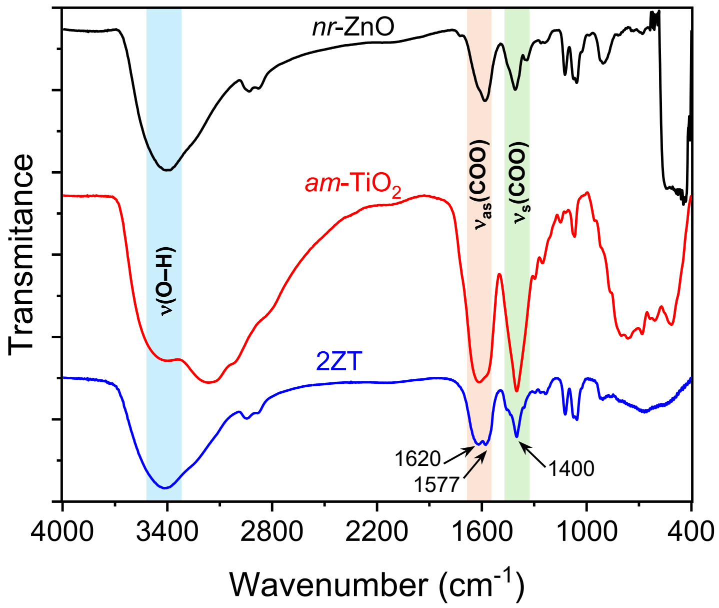

Figure S6. FTIR spectra of *nr*-ZnO, *am*-TiO_2, and 2ZT. The ν_{as} and ν_s represents asymmetric and symmetric stretching modes.

The FTIR spectra of *nr*-ZnO, *am*-TiO_2, and 2ZT were selected as representative because they allow clear comparison of Zn^{2+}–citrate interactions. The shift of the asymmetric COO^- stretching band from 1620 cm^{-1} in am-TiO_2 to 1577 cm^{-1} in 2ZT provides evidence of Zn^{2+} coordination to citrate ligands present on the TiO_2 surface. This shift, together with the increased intensity ratio of the 1577/1620 cm^{-1} bands, supports the proposed dissolution–adsorption mechanism in which citrate contributes to Zn^{2+} complexation, complementing

electrostatic effects. The peaks observed in the 1200–400 cm^{-1} range are typical of metal oxide nanoparticles and related materials. Such features are common but do not provide additional information relevant to the discussion presented in this work. For clarity of the spectrum and because they are not essential for our interpretation, these bands were not assigned.

Table S3. Corrected β parameter values and estimated crystallite sizes for the studied nanostructured materials.

Nanomaterials	β(100)/°	Size/nm	β(002)/°	Size/nm	β(101)/°	Size/nm
**ZnO**	0.357	23.16	0.176	47.27	0.353	23.72
**16ZT**	0.416	19.88	0.210	39.59	0.385	21.73
**8ZT**	0.421	19.63	0.214	38.97	0.386	21.67
**4ZT**	0.433	19.10	0.221	37.60	0.411	20.36
**2ZT**	0.420	19.68	0.240	34.67	0.432	19.34
**1ZT**	-	-	-	-	-	-

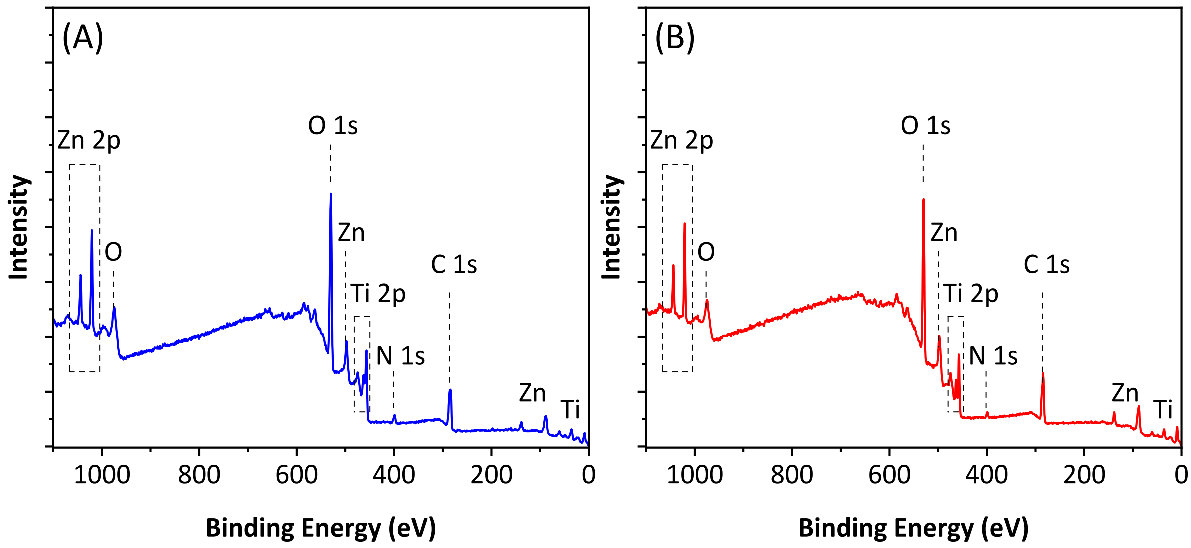

Figure S7. XPS survey spectra of (A) 1ZT, and (B) 8ZT samples.

Table S4. Estimated proportions of Ti(III) and Ti(IV) from XPS spectral peak deconvoluted areas in nanostructured materials considering the presence of Ti(III) sites.

Material	Proporção Ti(III)/Ti(IV)
P25	0
am-TiO_2	0.47
1ZT	0.82
8ZT	1.69

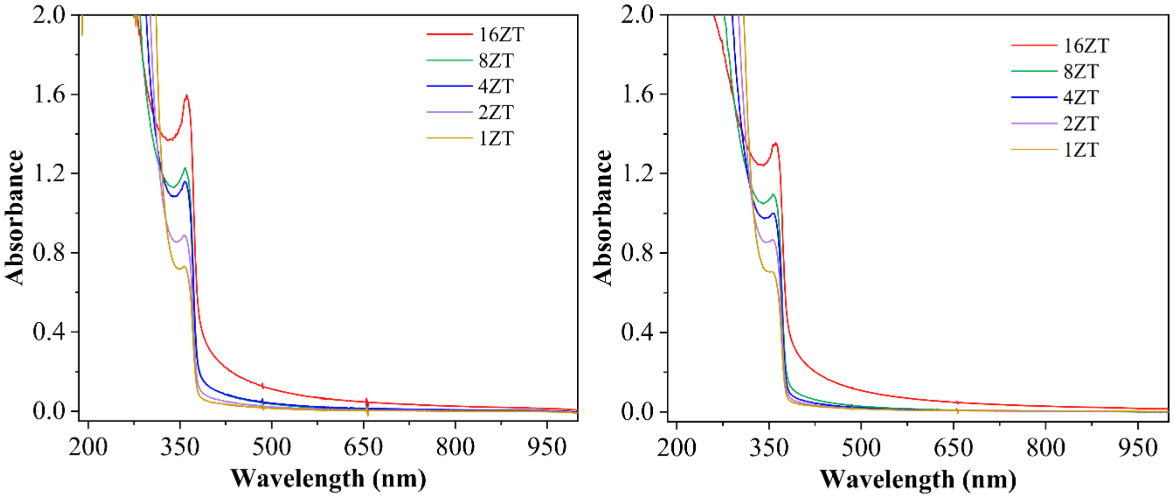

Figure S8. UV-Vis spectra of xZT nanocomposites prepared from two distinct samples of *nr*-ZnO mixed with different concentrations of *am*-TiO_2 NPs at room temperature.

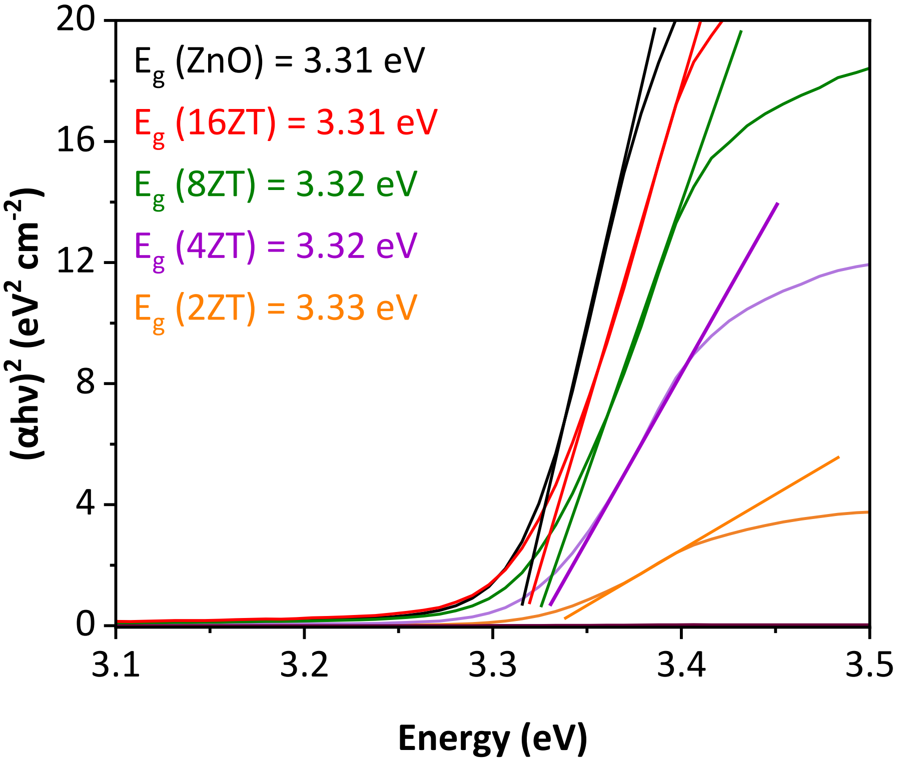

Figure S9. Tauc plot method applied to the nanomaterials.

Our findings differ from previous studies on ZnO–TiO_2 composites, which emphasized photocatalytic activity enhancement due to heterojunction formation. Here we demonstrate that amorphous ultrasmall TiO_2 induces ZnO size reduction by adsorption-driven dissolution. Future work will focus on evaluating photocatalytic and electronic properties of the size-adjusted ZnO@TiO_2 nanostructures and exploring the extension of this strategy to other oxide systems.

In comparison with previous reports that mainly focused on photocatalytic enhancement in TiO_2–ZnO composites, the present work emphasizes the unique dissolution–adsorption mechanism responsible for ZnO size reduction. Future studies will address the photocatalytic activity of these nanostructures in dye degradation and related processes, aiming to correlate size reduction with functional performance.

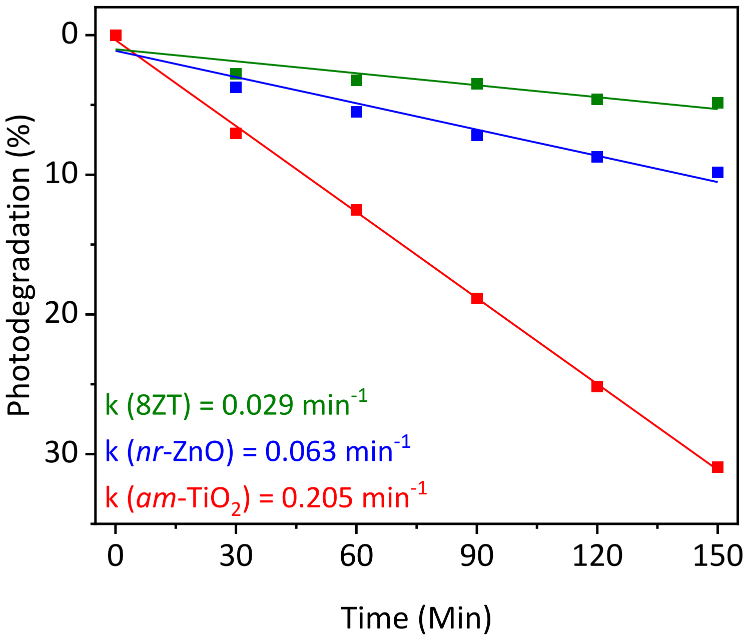

Figure S10. Photocatalytic degradation curves of Congo red aqueous solution (17.6 μmol L^{-1}) over time, using various nanomaterials at a concentration of 1.54 ppm.
